# Spatiotemporal Hotspots of Study Areas in Research of Gastric Cancer in China Based on Web-Crawled Literature

**DOI:** 10.3390/ijerph18083997

**Published:** 2021-04-10

**Authors:** Zhen Wang, Hongyan Ren, An Zhang, Dafang Zhuang

**Affiliations:** 1 State Key Laboratory of Resources and Environmental Information System, Institute of Geographic Sciences and Natural Resources Research, Chinese Academy of Sciences, Beijing 100101, China; wangz.16b@igsnrr.ac.cn (Z.W.); zhangan@igsnrr.ac.cn (A.Z.); 2 College of Resources and Environment, University of Chinese Academy of Sciences, Beijing 100190, China

**Keywords:** gastric cancer, study areas hotspots, space-time, toponym extraction, web-crawled literature, China

## Abstract

Gastric cancer is a common malignancy worldwide and poses a serious threat to human public health. The difficulty in obtaining epidemiological data limits the development of cross-disciplinary related research. In this study, 99,364 publications on gastric cancer from 1991 to 2019 were obtained using web-crawler technology, and a technical framework for extracting toponyms from these publications was constructed to analyze spatiotemporal hotspots of study areas in gastric cancer research in China. The results showed the following: (1) The accuracy of toponym extraction was greatly improved after eliminating the systematic exclusion words and adding historical toponyms, with a precision of 95.31% and a recall of 94.86%. (2) Gastric cancer research (GCR) and gastric cancer research with toponyms (GCRWT) are attracting increasing amounts of attention. The amount of GCR results published in Chinese and English is gradually leveling off, and the imbalance between those of GCRWT is gradually widening. (3) The spatial distribution of gastric cancer research in China is uneven, and the hotspots are mainly located in the eastern coastal areas. There were huge advances in gastric cancer research at the province/city/county scale in Eastern China, while the central region has only increased research at the county scale. We suggest that gastric cancer research should pay more attention to the central region, which has the highest gastric cancer incidence/mortality. This study provides important clues for research on and investigations of gastric cancer.

## 1. Introduction

Gastric cancer (GC) is a common malignancy worldwide, and it caused about 0.8 million deaths in 2018 [1]. China is one of the countries with the highest GC incidence and mortality, accounting for about 50% of new cases and deaths in the world, making it a major threat to the life and health of the Chinese people [2,3].

Research on the epidemiology, risk factors, and prevention and control strategies for gastric cancer based on surveillance data or statistical data is the basis and an important means of preventing and controlling gastric cancer [4,5]. China has had a sound national tumor registration system and a Disease Surveillance Points system (DSP) for decades, and the country has accumulated long-term tumor incidence and cause-of-death registration data [6,7]. However, for non-health research institutions in China, it is still difficult to obtain these epidemiological data, which to some extent affects the development of cross-disciplinary research (e.g., health and environment) and limits the possibility of disease prevention and control work being supported by theories and methods from more disciplines.

At present, the strategy for obtaining epidemiological data from the literature is a compromise for solving this problem, in which a meta-analysis based on published literature is a common method [8,9]. For example, Lewington et al. [10] used meta-analysis to study the age-related correlation between blood pressure and specific mortality and confirmed that blood pressure values in the middle-aged and elderly are closely related to vascular mortality. Wang et al. [11] used meta-analysis to study the prevalence of Helicobacter pylori (HP) infections in China and confirmed that HP is a risk factor for gastrointestinal diseases. Meta-analysis typically concerns the quantitative evidence-based analysis of different datasets and methods from the perspective of risk factors for many health problems. It often neglects the geographical location information of the research objects in the publications. However, because they are affected by the regional natural environment, social and economic development, and other factors, disease outbreaks and epidemics often have geographic spatial differences [12,13,14].

Research that considers geographical information in the literature can effectively mine and utilize the spatiotemporal distribution information of specific research themes, which can help researchers and government officials quickly and deeply grasp the characteristics of the current spatiotemporal pattern of the research, thus promoting the development of related research. For example, Hu et al. [15] mapped the spatial distribution of China’s rocky desertification research hotspots based on the literature and found that they differ from the sensitive areas of rocky desertification in terms of time, space, and concepts. Then, they provided suggestions as to the pertinence of the planning, governance, and study of rocky desertification. Liang et al. [16] analyzed the spatial distribution and dynamic changes in desertification research hotspots in China based on Chinese literature. They found the main distribution areas and that there is a declining trend in the research, which provides clues for the governance of desertification. However, similar studies applicable to the field of public health have not been carried out. Further, the related studies above did not discuss the accuracy of toponym extraction in detail, and the literature coverage needs to be improved.

Based on the above, this study used a web crawler to automatically and comprehensively obtain literature (written in Chinese and English) on gastric cancer research in China from the China National Knowledge Infrastructure (CNKI) and Web of Science (WOS). We constructed a toponym extraction framework from the literature and explored the spatiotemporal characteristics of gastric cancer research hotspots in China. The aim of this study was to provide an alternative method or tool for relevant institutions or scholars in the face of data limitations and to provide clues and a scientific reference for follow-up research on the epidemiology of gastric cancer.

## 2. Materials and Methods

### 2.1. Data Source

The basic literature databases for extracting the geographic spatial distribution information on gastric cancer research are the CNKI and WOS. The CNKI has developed into the world’s largest Chinese knowledge engine and resource site since the 1990s, with a total literature volume of more than 11,402 journals and 80 million articles [17]. The WOS is the most authoritative scientific and technological literature indexing tool in the world, covering more than 8700 core academic journals and 90 million articles [18]. The CNKI and WOS basically include all of the gastric cancer research literature in China. Therefore, in this study, journal articles related to gastric cancer published from 1991 to 2019 in CNKI and WOS were retrieved using a web crawler (see Section 2.2.1). Eliminating duplicates and irrelevant literature, 99,364 publications, including 78,104 Chinese publications in CNKI, and 21,260 English publications in WOS, were finally obtained. Then, the data were divided into six time stages, namely, 1991–1995, 1996–2000, 2001–2005, 2006–2010, 2011–2015, and 2016–2019, for the subsequent exploration of the spatial and temporal characteristics.

The Administrative Districts Data (2019) of China were collected from the Resource and Environment Science and Data Center, Chinese Academy of Sciences [19] and were used as the basic geographic data for the mapping and spatiotemporal analysis. The data for the standard and historical administrative district toponyms in China, which were obtained from the National Geomatics Center of China [20] and the National Bureau of Statistics of China [21], were used to standardize the toponyms extracted from the literature.

### 2.2. Methods

**Figure 1:**
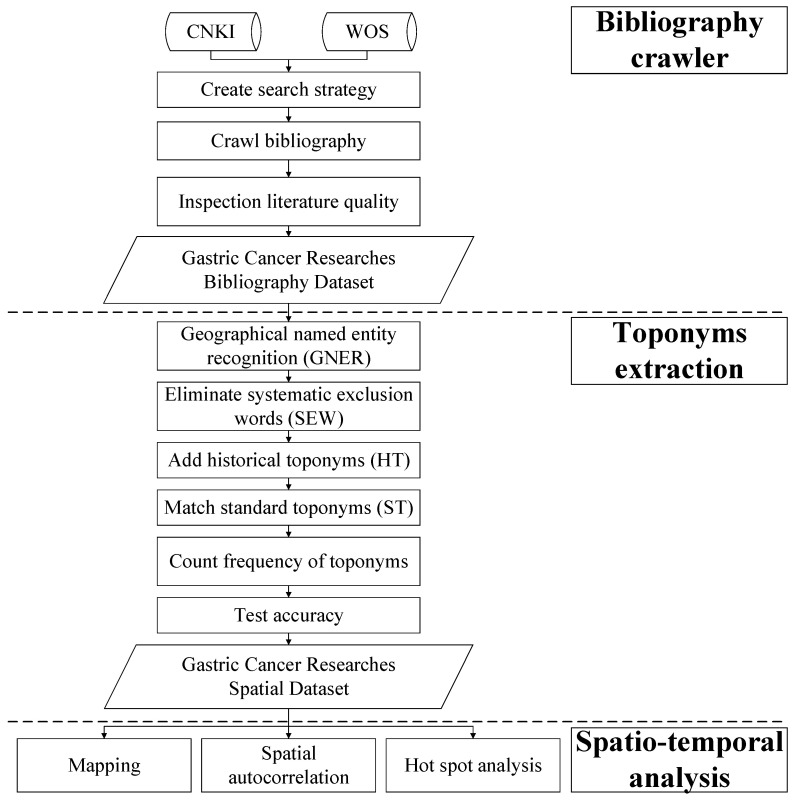
Overall workflow chart of the toponym extraction and spatiotemporal analysis of gastric cancer research hotspots in China.

This paper proposes a framework based on a literature bibliography crawler, geographic information extraction, and spatiotemporal analysis. Figure 1 shows the specific workflow chart.

#### 2.2.1. Bibliography Crawler

In order to realize automatic crawling of the CNKI and WOS literature bibliographies, in this study, first, a Chinese and English bibliographical information crawler was developed based on the powerful Scrapy crawler framework using the Python language [22]. The details of the crawler’s settings are shown in Table 1. Then, the CNKI and WOS databases were retrieved and crawled comprehensively. After this, the duplicate and irrelevant publications were removed. The key information retrieved, including the title, abstract, keywords, and publication time, was extracted and stored in the local database.

#### 2.2.2. Toponym Extraction and Accuracy Test

The Stanford CoreNLP toolkit, which has high accuracy in both Chinese and English geographical named entity recognition (GNER), was used to extract the toponyms in the titles, abstracts, and keywords of the publications [23]. We conducted a two-step post-proceed on the basis of the GNER (GNERPP). First, the error words of the toponym extraction from the literature, which are called systematic exclusion words (SEW) in this study, such as drug names containing toponyms (Table 2), were collected via sampling inspection, and an SEW database was established to clean all of the toponyms. Then, a toponym search lexicon [24] was established, including the standard toponyms (ST) and historical toponyms (HT), to standardize all of the toponyms and make them consistent with the Administrative Districts Data (2019) of China.

After cleaning the toponyms, an improved algorithm (i.e., the ascending scale toponym counting (ASTC) algorithm), based on the principle of ascending step by step and cumulative statistics, was built to count the occurrence frequency of the toponyms of the provinces/cities/counties [25]. In this process, first, we divided all of the ST into three levels: Province, city, and county. When we obtained a toponym from a publication after the GNERPP, we determined whether this toponym was at the province level. If it was, then the frequency of this toponym was increased. If not, we continued to determine whether the toponym contained a city-level toponym. If it did, we increased the frequency of the province-level toponym belonging to this toponym and itself. If matching still did not occur, we continued to determine whether the toponym contained a county-level toponym. If it did, we increased the frequency of the province-level and city-level toponyms belonging to this toponym and itself. In each step above, the duplicate toponyms in the same publication were identified and removed. The determination process continued on to the next toponym until all of the toponyms in the literature were processed.

In this study, the accuracy of the toponym extraction was tested using the recall and precision rate, which is commonly used in the text extraction accuracy test [26,27]. The uniform random sampling strategy was conducted as follows: One publication was randomly selected from every 40 publications for all of the 99,364 publications, and a total of 2600 samples, including 1998 Chinese samples and 602 English samples, were selected and labeled manually. The method of calculating the recall and precision rate is as follows:

(1) $$\;\;\;\;\;\;\;Recall=\frac{TP}{TP+FN}*100\%,$$

(2) $$Precision=\frac{TP}{TP+FP}*100\%.$$

where Recall stands for the recall rate of the toponym extraction; Precision stands for the precision of the toponym extraction; TP is the number of toponyms correctly extracted from the literature, which can be defined as Actual Positive $\cap^{\;}$ Predicted Positive; FN is the number of toponyms missed in the extraction that can be defined as Actual Positive $\cap^{\;}$ Predicted Negative; FP is the number of toponyms extracted incorrectly, which can be defined as Actual Negative $\cap^{\;}$ Predicted Positive.

#### 2.2.3. Spatial Processing and Analysis

In this study, Moran’s I was used to detect whether a significant spatial autocorrelation of gastric cancer research existed on the province, city, and county scales across China. The Moran’s I was calculated as follows:

(3) $$I=\frac{n\sum_{i=i}^{n}\sum_{j=1}^{n}\omega_{ij}\left(x_{i}-\bar{x}\right)\left(x_{j}-\bar{x}\right)}{\sum_{i=i}^{n}\sum_{j=1}^{n}\omega_{ij}\sum_{i=1}^{n}{\left(x_{i}-\bar{x}\right)}^{2}}.$$

where n is the number of administrative units; $x_{i}$ and $x_{j}$ are the number of gastric cancer studies in the administrative units i and j, respectively; $\bar{x}$ is the average value of the number of gastric cancer studies; and $w_{ij}$ is the spatial weight between administrative units i and j. The value of Moran’s I falls between −1 and 1 [28]. A statistically significant positive value indicates the presence of spatial clusters of gastric cancer studies, and adjacent administrative units have similar amounts of gastric cancer studies, whereas a statistically significant negative Moran’s I implies a tendency toward dispersal.

Based on the existence of spatial autocorrelation, the hotspot analysis was further utilized to capture the specific regions (provinces/cities/counties) with clustered high or low amounts of gastric cancer studies in China. $G_{i}^{*}$, which was proposed by Getis and Ord [29], was selected to identify the statistically significant gastric cancer research hotspots according to the following formula:

(4) $$G_{i}^{*}=\frac{\sum_{j=1}^{n}\omega_{ij}x_{j}-\bar{X}\sum_{j=1}^{n}\omega_{ij}}{S\sqrt{\frac{\left[n\sum_{j=1}^{n}\omega_{ij}^{2}-{\left(\sum_{j=1}^{n}\omega_{ij}\right)}^{2}\right]}{n-1}}}.$$

where $x_{j}$ is the number of gastric cancer studies in administrative unit j; $w_{ij}$ is the spatial weight between administrative unit i and j; n is the number of administrative units; and

(5) $$\bar{X}=\frac{\sum_{j=1}^{n}x_{j}}{n},$$

(6) $$S=\sqrt{\frac{\sum_{j=1}^{n}x_{j}^{2}}{n}-{\left(\bar{X}\right)}^{2}}.$$

The $G_{i}^{*}$ statistic is a Z-score. For statistically significant positive Z-scores, the larger the Z-score, the more intense the clustering of a large amount of gastric cancer studies (i.e., a hot spot). For statistically significant negative Z-scores, the smaller the Z-score, the more intense the clustering of a small amount of gastric cancer studies (i.e., a cold spot).

The construction of a spatial dataset for gastric cancer research, spatial autocorrelation, and hotspot analysis were conducted using the ArcGIS 10.2 platform (ESRI, USA).

## 3. Results

### 3.1. Accuracy of the Toponym Extraction

In order to test the accuracy of the toponym extraction, in this study, the recall and precision rate of the toponym extraction were compared with the GNER and GNERPP. As can be seen from Table 3, the precision of the toponym extraction, which used geographical named entity recognition (GNER), is only 73.66%, and the extraction precision increased to 95.31% with an improvement of 30% after the post-processing steps (GNERPP), i.e., the elimination of the SEW and the addition of HT. There was also a slight increase in the recall rate (from 90.19% to 94.86%). As can be seen, in this study, the extraction accuracy of the toponyms was relatively high after the elimination of the systematically excluded words and the addition of historical toponyms, and thus, it met the requirements of the subsequent spatiotemporal analysis.

### 3.2. Global Quantitative Characteristics

In this study, we called gastric cancer research (GCR) publications that contained toponyms gastric cancer research with toponyms (GCRWT). Most of the GCRWT focused on the epidemiology of gastric cancer in a certain administrative region of China and explored the impact of the natural, economic, and social factors on the incidence and prevalence of gastric cancer [30,31,32]. In order to explore the overall amount of attention paid to gastric cancer studies in the past three decades in China, in this study, the inter-annual variation in the GCR and GCRWT from 1991–2019 was analyzed.

**Figure 2:**
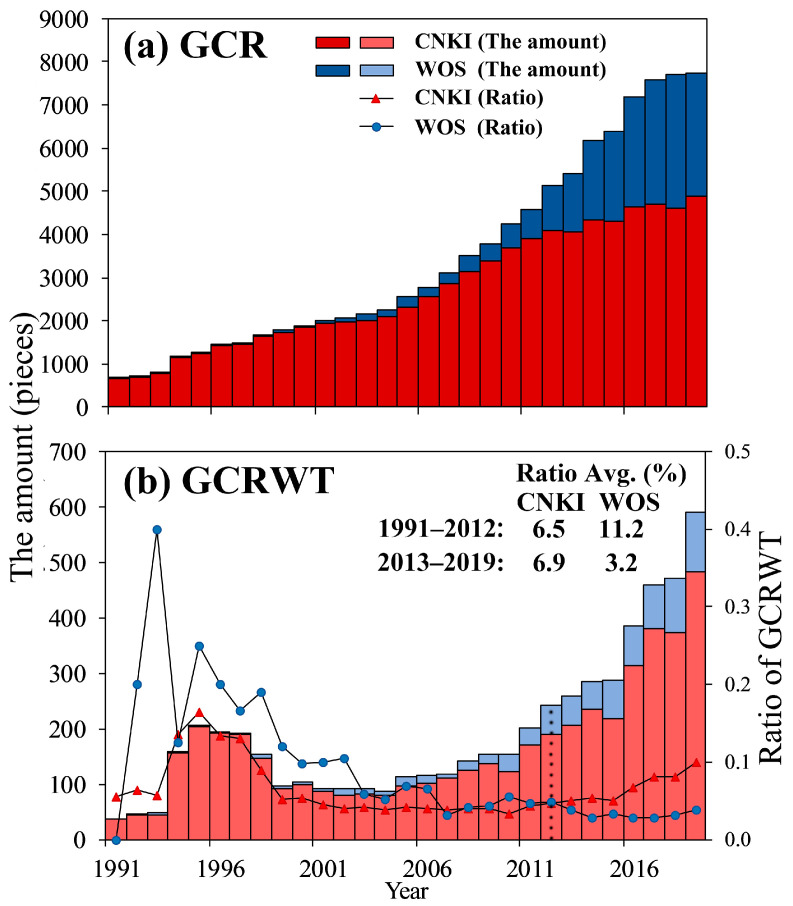
Interannual changes in the amount of gastric cancer research (GCR) and gastric cancer research with toponyms (GCRWT) (1991–2019). (**a**) The amount of GCR in the CNKI and WOS; (**b**) the amount of GCRWT and GCRWT ratio in the CNKI and WOS (GCRWT Ratio = GCRWT/GCR × 100%; Ratio Avg. is the average of the GCRWT Ratio by year, and the dotted line marks the dividing line of the GCRWT Ratio).

As can be seen from Figure 2, the amount of GCR in the CNKI and WOS databases exhibits a clear trend of rapid increase, with the proportion of the publications in the CNKI and WOS gradually leveling off. The amount of GCRWT in the CNKI and WOS also exhibits a rapid increase. However, the GCRWT in the CNKI was much larger than that in the WOS, and there was even a small upsurge in the early period (1993–2000). Similarly, the GCRWT rates (GCRWT/GCR × 100%) were also larger in the CNKI than in the WOS in recent years: WOS (11.2%) was larger than the CNKI (6.5%) from 1991 to 2012, and the CNKI (6.9%) was larger than the WOS (3.2%) from 2013 to 2019. As can be seen, the attention paid to gastric cancer research in China is increasing, and the research results published in Chinese and English are becoming more balanced year by year. Gastric cancer research with toponyms has also attracted increasing amounts of attention, but far more results are published in Chinese than in English, and the degree of imbalance has increased in recent years.

### 3.3. Spatiotemporal Characteristics

#### 3.3.1. Spatial Pattern

**Figure 3:**
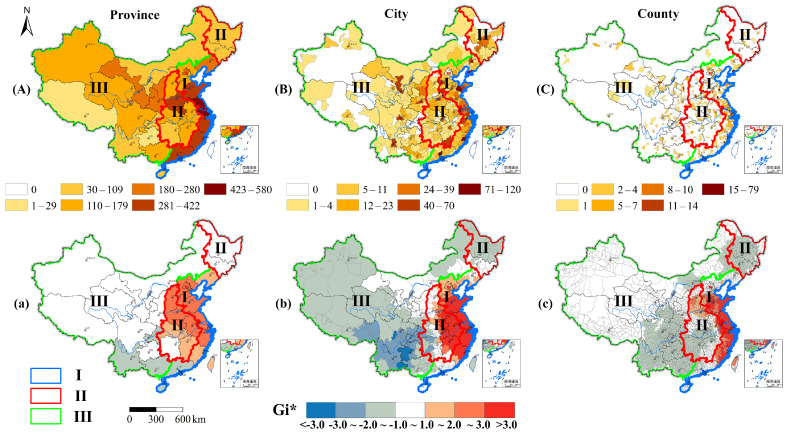
Spatial distribution of gastric cancer research and hotspots at the province, city, and county scales in China from 1991 to 2019. (**A**), (**B**), and (**C**) are the spatial distributions of the number of gastric cancer studies at the province, city, and county scales, respectively. (**a**–**c**) are the spatial distributions of gastric cancer research hotspots at the province, city, and county scales, respectively. (I: Eastern region; II: Central region; III: Western region [33]).

In this study, based on the characteristics of the units of gastric cancer research in China, we divided them into three-levels, i.e., the province, city, and county scales, to map and analyze the spatial distribution of the amount of research and the gastric cancer research hotspots in China. Thus, according to the division of the three regions (eastern region, central region, western region; their boundaries are shown in Figure 3.), which are commonly used in the epidemiological statistics of gastric cancer in China [5,33], the regional amount characteristics of gastric cancer research were also statistically analyzed.

**Figure 4:**
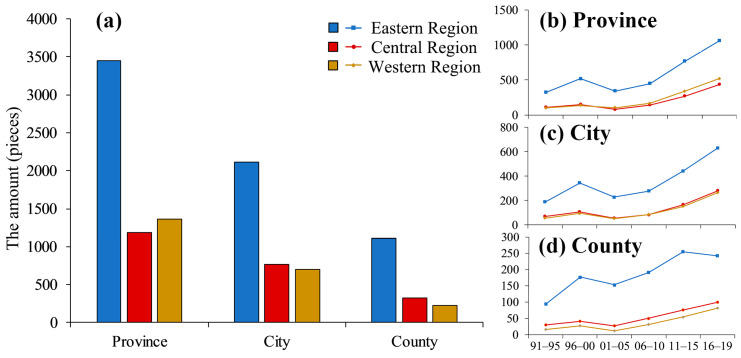
The number of gastric cancer studies in the eastern, central, and western regions at the province, city, and county scales and their changes in the different time periods. (**a**) The number of gastric cancer studies at the province, city, and county scales in the three regions; (**b**–**d**) the changes in the gastric cancer studies at the province, city, and county scales at different time periods in the three regions.

As is illustrated in Figure 3, at the province scale (Figure 3A), gastric cancer studies cover the entire country, and they are mainly distributed in the eastern coastal area, the Hexi Corridor, northeastern Liaoning, Shaanxi, and Henan (Table S1). The spatial distribution shows a significant spatial autocorrelation (Moran’s I = 0.186 ***) (Table 4). The research hotspots (Figure 3a) are mainly distributed in the eastern coastal areas and parts of the central region. The central region (Figure 4b) had the lowest amount of gastric cancer studies at the province scale (eastern region > western region > central region).

At the city scale (Figure 3B), gastric cancer studies basically cover the entire country, except for some remote areas, and they exhibit a dense and continuous zonal distribution characteristic with a significant spatial autocorrelation (Moran’s I = 0.118 ***) (Table 4). Most of the cities with larger amounts of gastric cancer studies are provincial capitals, but some non-provincial capital cities also fall within this category (Table S1). The distribution of the research hotspots (Figure 3b) is basically the same as at the province scale, but the Gi^*^ is larger. The number of studies on gastric cancer in the central and western regions is basically the same (Figure 4c) (eastern region > central region ≈ western region).

At the county scale (Figure 3C), gastric cancer studies generally exhibit a discontinuous point distribution characteristic, they are densely distributed in some provinces and cities, and a significant spatial autocorrelation also exists (Moran’s I = 0.048 ***) (Table 4). There are counties with concentrated distributions in the Yangtze River Delta, southern Fujian, and the Pearl River Delta (Table S1). The research hotspots at the county scale (Figure 3c) are distributed along the eastern coast and closer to the coast than on the province and city scales, and a small number of research hotspots are located around the Taihang Mountains in Henan and Shanxi provinces. The number of gastric studies is slightly higher in the central region than in the western region (Figure 4d) (eastern region > central region > western region).

The results showed that the spatial distributions of gastric cancer research at the provincial, city, and county levels in China were uneven. The research hotspots were mainly distributed in the eastern coastal areas. The eastern region had the highest amount of gastric cancer studies at all three scales, and the central region only had more studies at the county scale than the western region.

#### 3.3.2. Spatial and Temporal Changes

In this study, six time periods were mapped, and the autocorrelation and spatiotemporal changes in the gastric cancer research hotspots from 1991 to 2019 were analyzed. The studies at the province, city, and county scales showed significant spatial agglomeration in each time period (Table 4). At the province scale, the Moran’s I increased from 1991 (0.130) to 2015 (0.220) and then decreased in 2016–2019 (0.117). At the city scale, it stably increased from 1991 (0.064) to 2019 (0.115). At the county scale, it began with a large value (0.048) in 1991–1995, stably increased from 1996 (0.017) to 2015 (0.052) and remained stable in 2016–2019 (0.050).

**Figure 5:**
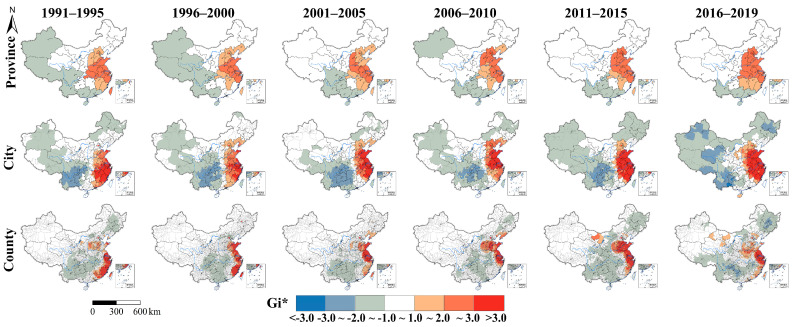
Spatiotemporal distribution of the gastric cancer research hotspots in China in the six time periods from 1991 to 2019 at the province, city, and county scales. The study period was divided into six time periods: 1991–1995, 1996–2000, 2001–2005, 2006–2010, 2011–2015, and 2016–2019.

As is illustrated in Figure 5, there were obvious gastric cancer research hotspots at the province, city, and county scales in China in each time period, which were mainly distributed in the eastern coastal areas, especially the stable hotspots located in the Shandong Peninsula, Yangtze River Delta, and Huaihe River Basin. These results showed that the gastric cancer research exhibited uneven spatiotemporal distributions at the provincial, city, and county scales in China during the different time periods from 1991 to 2019, and the eastern coastal region continuously contained most of the hotspot areas.

## 4. Discussion

Gastric cancer is a serious threat to human public health, and epidemiological methods are an important means of studying and solving the problems related to gastric cancer. However, it is difficult for researchers in non-health fields to obtain epidemiological data, which limits the development of interdisciplinary research on health and the environment. In this study, a framework for the accurate extraction of toponyms from the literature was established, and the spatiotemporal characteristics of the gastric cancer research hotspot areas in China were analyzed. The method developed in this study provides a new solution for obtaining data related to gastric cancer and provides a scientific reference for subsequent studies of the epidemiology of gastric cancer.

This study covered the gastric cancer research literature more comprehensively, achieved a higher toponym extraction accuracy, and carried out spatiotemporal analysis at the provincial, city, and county scales, which is more suitable for gastric cancer research in China. The previous studies [16,17] that chose CNKI as data source mainly covered the literatures in Chinese, and used the toponym counting algorithm called “Descending scale counting”, which accumulated occurrence frequency of toponyms from province/city level to county level. By contrast, our study increased the coverage of literatures and improved toponyms extraction and counting algorithm for gastric cancer research. First, the CNKI and WOS were used to comprehensively cover the literature published in both Chinese and English. Second, the GNERPP was carried out in which the SEW were eliminated and HT were added in the extraction process to make the toponyms extracted more accurate than in past studies that used the GNER [15,16,34]. Third, the precision and recall rate were tested to quantitatively analyze the accuracy of the toponym extraction in order to ensure the correctness of the subsequent analysis. In addition, the research and prevention-control of diseases in China, especially gastric cancer, are always carried out using administrative regions (provincial, city, and county scales) as units [32]. This study proposes an ASTC method of counting the toponyms for the analysis of three-levels of administrative regions, which are more suitable for studies of gastric cancer than that used in past studies [15].

In the context of increasing attention in China, the publication of GCRWT results in English needs to be strengthened. Currently, in China, the publication of foreign language research results and the development of international cooperation can promote international exchange and the in-depth development of research in all fields [35]. Therefore, we believe that the GCRWT in China needs to be strengthened in terms of the foreign-language publication of research results to increase international exchange and the in-depth development of gastric cancer research. On the one hand, the reason for this is that we can see China pays more and more attention to GCR, and thus, the publication of studies in Chinese and foreign languages are becoming more balanced year by year. On the other hand, although the attention to GCRWT is also increasing, the imbalance between the Chinese and English GCRWT still exists, and the gap between them has gradually widened in recent years.

There are differences between the geographic spatial distribution of the high incidence areas and gastric cancer research hotspots. In other words, the gastric cancer research hotspots may not be the prevalent hotspots of gastric cancer, and this difference may have a dual impact on the incidence/mortality of gastric cancer and the level of economic development. First of all, previous studies have shown that northern Xinjiang, the Hexi Corridor, Ningxia, northern Shaanxi, Inner Mongolia, Liaoning, the Jiaodong Peninsula, Jiangsu, Zhejiang, and Fujian are the main regions with a high prevalence of gastric cancer in China [36,37,38], which is basically consistent with research hotspots found in this study at the province and county scales. However, a small number of studies at the province and city scales are located in low gastric cancer incidence areas, such as economically developed Guangdong Province and the provincial capitals of Chengdu and Kunming. Therefore, the incidence/mortality of gastric cancer and the level of economic development may affect the attention paid to gastric cancer research on the epidemiology and economy levels, which results in the different spatial distribution between the epidemic and research hotspots of gastric cancer in China.

The spatiotemporal distribution of gastric cancer research in China is uneven, and areas with high incidences but no research hotspots should receive more attention in the future. The eastern region of China had a great advantage in gastric cancer research at all three scales in all of the time periods from 1991 to 2019; whereas the central region only had more studies at the county scale. The possible reasons for this are as follows. First, the eastern region of China has a relatively high level of economic development, so more attention and resources can be devoted to gastric cancer research, which seriously affects the health of the residents of this area [30]. Second, compared with provincial- and city- scale studies, county-scale gastric cancer research require fewer resources to acquire experimental data, which gives researchers in county regions more choices and chances to carry out epidemiologic studies on gastric cancer [31,39]. Third, since the 1990s, several typical study sites have been set up in high incidence areas of gastric cancer with the help of the Chinese government, such as Linzhou City and Linqu County [40,41], providing a great deal of epidemiologic data, which support gastric cancer studies in these county regions. However, according to the 2019 China Cancer Report, the age-standardized incidence/mortality rate of gastric cancer in the central region (22.00/10^5^) is much higher than in the eastern (17.66/10^5^) and western (15.81/10^5^) regions of China [5]. Therefore, we suggest that increasing attention should be paid to gastric cancer research in the central region of China, where the level of economic development is lower, but the incidence/mortality of gastric cancer is the highest.

This study used the timing of the publications, which may be different from the timing of the research data. This study focused on the timing of gastric cancer studies, so the usage of the publication time could be appropriate for this study. While there may be a lag in the publication of research results based on epidemic data of gastric cancer and other cancers [3,5], there may also be a lag in the reflection of gastric cancer research hotspots on areas with high incidence of gastric cancer.

There are several limitations to this study. First, more databases such as PubMed, Taylor, Wanfang, and VIP should be added to further improve the coverage of the Chinese and English literature. Second, studies on the influencing factors of research hotspots in combination with other environmental data (pollution and governance, social and economic development, living habits, etc.) should be carried out. Third, the extraction of numerical indicators (incidence/mortality, OR/RR value of risk factors, etc.) could be added to the text extraction framework based on literature to obtain and mine more useful information. Finally, a research hotspot index model based on the literature should be established to further quantitatively measure the attention to diseases research in a future study.

## 5. Conclusions

In this study, an accurate geographic information extraction framework based on authoritative and professional scientific research publications was established. It is suitable for analyzing the spatiotemporal characteristics of research hotspots for gastric cancer and other diseases. Regional imbalances exist in gastric cancer research hotspots in China. We suggest that more attention should be paid to gastric cancer research in the central region of China. This study provides an important reference for the investigation of gastric cancer.

## Figures and Tables

**Table 1:** Bibliography crawler settings for the China National Knowledge Infrastructure (CNKI) and Web of Science (WOS) databases.

Database	Search Strategy	Request (accessed on 4 May 2020)	Parsing Method	Remark
CNKI	SU = ‘wei ai’ OR (SU = ‘wei’ AND SU = ‘e xing zhong liu’)	**Search:** http://kns/request/searchhandler.ashx **Results:** http://kns.cnki.net/kns/brief/brief.aspx **Result Details:** http://kns.cnki.net/kns/detail/detail.aspx	Beautiful Soup, Regular expression	Crawl year by year because the number of search results is limited for a single time (less than 5000)
WOS	(TS = gastric cancer OR TS = cancer of the stomach OR TS = gastric carcinoma) AND CU = CHINA	**Search:** http://apps.webofknowledge.com/WOS_AdvancedSearch.do **Results:** http://apps.webofknowledge.com//OutboundService.do?action=go&&	Automatic export	Export every 500 pieces, using the automatic export function

**Table 2:** Examples of systematic exclusion words (SEW) and historical toponyms (HT).

GNERPP	Text in the Literature	Mis-Extraction Result (SEW)/Standard Toponym (HT)
SEW	Yunnan Baiyao	Yunnan Province
	Pingyang Meisu	Pingyang County, Wenzhou City, Zhejiang Province
	Shanghai Ruijin Hospital	Ruijin City, Ganzhou City, Jiangxi Province
	Zhongshan Hospital of Fudan University	Zhongshan City, Guangdong Province
	Gansu Hexi	Hexi District, Tianjin
	...	...
HT	Xiangfan City	Xiangyang City, Hubei Province (2010)
	Linxian County	Linzhou City, Anyang City, Henan Province (1994)
	Changle County	Changle City, Fuzhou City, Fujian Province (1994) Changle District (2017)
	Chongwen District ...	Dongcheng District, Beijing (2010) ...

**Table 3:** Confusion matrix of the toponym extraction.

		Actual (GNER)				Actual (GNERPP)			
		Positive	Negative	Sum	Precision (%)	Positive	Negative	Sum	Precision (%)
Predicted	Positive	193	69	262	73.66	203	10	213	95.31
	Negative	21	2317	2338	/	11	2376	2387	/
Sum		214	2386	2600	/	214	2386	2600	/
Recall (%)		90.19	/	/	/	94.86	/	/	/

**Table 4:** Spatial autocorrelation of the gastric cancer studies in China at three scales in each time period.

Time Stage	Province			City			County		
	Moran’s I	Z-Score	*p*-Value	Moran’s I	Z-Score	*p*-Value	Moran’s I	Z-Score	*p*-Value
91–95	0.130 **	2.063	0.039	0.064 ***	6.850	<0.001	0.048 ***	13.976	<0.001
96–00	0.159 **	2.409	0.016	0.062 ***	6.890	<0.001	0.017 ***	5.594	<0.001
01–05	0.155 **	2.460	0.014	0.074 ***	7.994	<0.001	0.031 ***	9.492	<0.001
06–10	0.176 ***	2.630	0.009	0.081 ***	8.599	<0.001	0.021 ***	6.454	<0.001
11–15	0.220 ***	3.181	0.001	0.108 ***	11.068	<0.001	0.052 ***	15.240	<0.001
16–19	0.117 *	1.841	0.066	0.115 ***	11.749	<0.001	0.050 ***	14.269	<0.001
91–19	0.186 ***	2.719	0.007	0.118 ***	12.072	<0.001	0.048 ***	14.229	<0.001

Note: *, **, and *** indicate that the spatial autocorrelation is significant at the 0.1, 0.05, and 0.01 confidence interval, respectively.

## Data Availability

The data presented in this study are available on request from the corresponding author. The data are not publicly available due to provide private access.

## Supplementary Materials

The following are available online at https://www.mdpi.com/article/10.3390/ijerph18083997/s1, Table S1: Areas with more gastric cancer studies at the province, city, and county scales in each time period.

## Abbreviations

GC	Gastric Cancer
GCR	Gastric Cancer Research
GCRWT	Gastric Cancer Research with Toponyms
GNER	Geographical Named Entity Recognition
GNERPP	Post-proceed after Geographical Named Entity Recognition
SEW	Systematic Exclusion Words
ST	Standard Toponyms
HT	Historical Toponyms
ASTC	Ascending Scale Toponym Counting
