# Supplementary material for: Spatiotemporal Hotspots of Study Areas in Research of Gastric Cancer in China Based on Web-Crawled Literature
Source: Int J Environ Res Public Health. 2021 Apr 10;18(8):3997. doi: 10.3390/ijerph18083997 (PMC8070457; doi:10.3390/ijerph18083997)
Supplement: Supplementary file 1 [file ijerph-18-03997-s001.pdf]

Table S1. Areas with more gastric cancer studies at the province, city, and county scales in each time period.

Time Stage	Province			City			County		
	Eastern Region	Central Region	Western Region	Eastern Region	Central Region	Western Region	Eastern Region	Central Region	Western Region
1991–1995	Shanghai, Fujian, Jiangsu	/	/	**Fuzhou, Nanjing, Guangzhou,** Dalian, **Shijiazhuang**	/	/	Changle District, Linqu, Zanhuang, Zhuanghe	Linzhou	/
1996–2000	Shanghai, Fujian, Shandong	/	/	**Fuzhou, Guangzhou, Shenyang,** Linyi, Weifang	/	/	Changle District, Linqu, Lanling, Yangzhong, Zanhuang	/	/
2001–2005	Jiangsu, Shanghai, Fujian	/	/	**Fuzhou,** Taizhou, Dalian, Weifang	/	Wuwei	Changle District, Taixing, Linqu, Yangzhong	Linzhou	/
2006–2010	Jiangsu, Shandong, Shanghai	/	/	Weifang, Dalian, **Nanjing**	Anyang	Wuwei	Linqu, Zhuanghe, Cixian, Changshu	Linzhou	/
2011–2015	Jiangsu, Shanghai	Henan	/	**Nanjing**, Weifang, **Guangzhou**	Anyang	Wuwei	Linqu, Cixian, Shexian	Linzhou	Liangzhou District
2016–2019	Jiangsu, Guangdong	Henan	/	**Nanjing, Guangzhou**	**Zhengzhou, Wuhan**	**Xi 'an**	Xianyou, Cixian, Zhangjiagang, Yangzhong	Linzhou	/
1991–2019	Jiangsu, Shanghai, Shandong, Zhejiang, Fujian, Guangdong, Beijing, Liaoning, Hebei	Henan, Hubei	Gansu, Shaanxi, Sichuan, Qinghai	**Fuzhou**, Weifang, **Nanjing,** Dalian, **Guangzhou,** Suzhou, Qingdao, Ningbo, **Hangzhou, Kunming**	Anyang, **Wuhan, Zhengzhou**	Wuwei, **Xi'an**	Linqu, Changle District, Yangzhong, Zhuanghe, Cixian, Taixing, Zanhuang, Lanling, Shexian, Kunshan, Qidong, Changshu, Xianyou	Linzhou, Yangcheng	/

Note: six time periods (province: TOP 3; city and county: TOP 5); 1991–2019 (province, city, and county: TOP 15). The provincial capitals are marked in bold.
